# Pharmacological Modulation of Oxidative Stress

**DOI:** 10.3390/ijms241914455

**Published:** 2023-09-22

**Authors:** Sarmistha Saha, Luciano Saso

**Affiliations:** 1 Department of Biotechnology, Institute of Applied Sciences & Humanities, GLA University, Mathura 281406, Uttar Pradesh, India; sarmistha_pharmacol@yahoo.com; 2 Department of Physiology and Pharmacology “Vittorio Erspamer”, Sapienza University of Rome, 00185 Rome, Italy

## Introduction

An imbalance between the formation of reactive oxygen species (ROS) and the reaction of antioxidant proteins is referred to as oxidative stress. Cellular signalling, differentiation, autophagy, and metabolic adaptability are all physiological processes that are aided by enzymatic and non-enzymatic antioxidants, which keep the ROS at low levels [1]. Oxidative stress is a condition in which ROS overwhelms the cellular antioxidant capability. ROS overproduction can potentially harm macromolecules necessary for life, including lipids, nucleic acids, and proteins [2]. This cumulative harm can cause cellular senescence or malfunction, which can harm organs and result in several degenerative disorders, including cancer [3]. Oxidative stress may affect various biological processes, including the acceleration of all pathological mechanisms, such as inflammation, apoptosis, dysregulation of autophagy, mitochondrial dysfunction, and endoplasmic reticulum stress [4]. Antioxidants’ function has undergone a considerable redefinition during the past 20 years. Numerous epidemiological and clinical investigations have questioned the presumption that “classical antioxidants” (radical scavengers) are always beneficial for maintaining human health. It took some time to realise that small compounds can precisely alter the activities of proteins involved in the antioxidant system to provide a more notable antioxidant impact [5]. In light of these drawbacks, a more advantageous strategy would involve focusing on the oxidative stress-induced signalling pathways implicated in the pathogenesis of illness or the control of metabolic enzymes and efflux transporters. It is essential to comprehend how oxidative stress and underlying pro- and anti-oxidant systems interact to comprehend disease mechanisms and identify and create novel, precise therapeutic approaches. Oxidative stress activates transcription factors such as the Nrf2, NF-κB, and AP1 [6].

The transcription factor Nrf2 (NF-E2-related factor 2), a member of the Cap’n’collar (CNC) family, and its antagonistic regulator, Kelch-like ECH-associated protein 1 (Keap1), are among the key antioxidant systems [7]. Normal circumstances result in Keap1 binding to Nrf2’s Neh2 domain through the ETGE and DLG motifs, leading Nrf2 to localise in the cytoplasm. In contrast, oxidative stress causes the E3–ligase complex to change conformation, which prevents Nrf2 from engaging with the ubiquitin-conjugating process [7]. The Nrf2 is released from the complex and moves into the nucleus, forming a heterodimer with the sMaf protein before being activated by the ARE. The ARE further regulates the synthesis of antioxidant proteins and cell defence mechanisms [8]. A significant area of medical attention will continue to be the development of Nrf2-targeted therapies. It has been emphasised that the activation of the Nrf2 signalling pathway can be used as a pharmaceutical strategy to systematically reduce the damage caused by oxidative stress in several chronic disorders. So far, dimethylfumarate (DMF), a fumaric acid ester, is the only drug approved by the FDA as an Nrf2 activator for treating patients with multiple sclerosis [9]. Because of their structural variety, tolerance, safety profiles, and pharmacokinetic characteristics, significant progress has been achieved in the synthesis of small compounds as well as the investigation of natural derivatives for Nrf2 activation; however, subsequent studies are needed to assess the absorption, metabolism, and excretion properties [10]. Therefore, for meaningful clinical applications of Nrf2 activators that could result in clinical applications, it may be more logical to design therapeutics based on the target selectivity in the relevant tissues. This Special Issue shares insights on the pharmacological aspects of research on oxidative stress in different pathological scenarios, with particular attention paid to the activation of the Nrf2 signalling pathway.

The study by Tucci et al. [11] documents the role of Nrf2 in Huntington’s disease. Another study discussed the NRF2 activators that may be used as a preventative measure or a treatment for HD neurodegeneration. OS is mostly produced due to an imbalance between ROS, RNS, and antioxidant molecules, which is primarily mediated by neurons and microglia [12]. Unsaturated lipids, Fe^2+^ or Cu^+^, are a perfect trigger for cytotoxic lipid peroxidation in the brain [13]. Another study summarised the recent studies relating to 2-Cyano-3,12-Dioxooleana-1,9-Dien-28-Oicaci-methyl amide (CDDO-MA), CDDO-ethyl amide (CDDO-EA) and CDDO-trifluoroethyl amide (CDDO-TFEA), which reportedly enhanced motor function, reduced oxidative stress, and increased transcription of genes controlled by Nrf2/ARE [14]. Another study discussed luteolin and its four synthetic derivatives Lut-C1, Lut-C4, Lut-C6, and Lut-C10), ginsenosides dammarane type triterpene glycosides, gintonin as a lysophosphatidic acid receptor (LPARs) ligand, fullerene C60 and 2,4-diamino-6-hydroxypyrimidine (DAHP) for the neutralization of ROS and activation of Nrf2 pathway in neurodegeneration [15,16]. Besides these, synthetic derivatives of natural products such as diapocynin, an oxidative derivative of apocynin, and harmine, a plant-derived carboline alkaloid, were shown to decrease the aggregation of mutant huntingtin protein (mHTT) and oxidative stress with the activation of Nrf2 protein, and thereby restored redox homeostasis [17,18].
